# F181. CHANGE IN PREFRONTAL-LIMBIC MORPHOLOGY AND COGNITION IN DRUG-NAïVE FIRST-EPISODE PSYCHOSIS PATIENTS FOLLOWING ATYPICAL ANTIPSYCHOTIC TREATMENT: A BRIEF LONGITUDINAL STUDY

**DOI:** 10.1093/schbul/sby017.712

**Published:** 2018-04-01

**Authors:** Melissa Woodward, Geoffrey Smith, William Honer, Vina Goghari, Lili Kopala, Kristina Gicas, Allen Thornton, Wayne Su, William MacEwan, Donna Lang

**Affiliations:** 1 University of British Columbia; 2 University of Toronto; 3 Simon Fraser University

## Abstract

**Background:**

Atypical antipsychotics are thought to normalize structural morphology in subcortical regions, however their effect on cortical volume remains equivocal.1,2 Studying the impact of atypical antipsychotic treatment on cortical structure in drug-naïve first-episode psychosis (FEP) patients is an opportunity to elucidate the effects of illness chronicity and treatment. Previous work has indicated the potential for short-term atypical antipsychotic treatment to increase cortical thickness in FEP patients, particularly the rostral and caudal middle frontal cortices.3 Both entorhinal and orbitofrontal cortices are decreased in patients with schizophrenia and impairment in prefrontal-limbic circuitry has been linked with cognitive impairment in patients.4,5 We examined the ability of an eight-week atypical antipsychotic treatment to increase entorhinal cortex (ERC) and orbitofrontal cortex (OFC) volume and thickness and improve symptom severity in drug-naïve FEP patients.

**Methods:**

Twenty-three FEP patients treated with risperidone or quetiapine and 28 healthy volunteers completed structural 3T magnetic resonance imaging, neurocognitive testing and clinical assessments at baseline, four weeks and eight weeks. Volumetric segmentation of the cortical regions of interest was performed with Freesurfer 5.3 software. Baseline and eight-week follow-up assessments were used to calculate change scores for clinical, cognitive and structural variables to compare between groups. Change in volume, clinical and cognitive scores were analyzed with ANCOVA with age, antipsychotic dose and total brain volume entered as covariates.

**Results:**

FEP patients and healthy volunteers did not differ significantly in volume or thickness for both ERC and OFC regions at baseline. FEP patients demonstrated a significant increase in OFC volume (F(22,1)=5.23, p=0.34) and an increase in ERC thickness (F(22,1)=12.80, p=0.002) following treatment. Healthy volunteers had an increase in ERC volume (F(27, 1)=4.99, p=0.35). Cognitive switching, an indicator of executive functioning, was not predicted by our brain measures of interest at baseline. Following treatment, increased OFC volume predicted a worse performance on the cognitive switching task for patients (β(22,1)=0.770, p=0.017) but a better score for healthy volunteers (β(23,1)=-0.712, p=0.044). Symptom severity scores were not significantly related to our brain regions of interest.

**Discussion:**

FEP patients have increase entorhinal cortical thickness and orbitofrontal cortical volume following an 8-week treatment of atypical antipsychotics. Increased OFC volume is associated with a decreased proficiency at cognitive switching for FEP patients but an increased proficiency for healthy volunteers. This difference may be due to underlying neurodevelopmental differences between psychosis patients and healthy controls and improvement in neurocognitive tasks may occur given a longer duration of antipsychotic treatment. These findings demonstrate the impact of atypical antipsychotics on cortical morphology in key regions associated with psychosis-spectrum disorders.

